# Supplementary material for: The causal relationship between the human gut microbiota and pyogenic arthritis: a Mendelian randomization study
Source: Front Cell Infect Microbiol. 2024 Nov 26;14:1452480. doi: 10.3389/fcimb.2024.1452480 (PMC11629706; doi:10.3389/fcimb.2024.1452480)
Supplement: Supplementary file 1 [file DataSheet1.zip › mendelian test/ebi-a-GCST90027668.csv_leave_one_out.pdf]

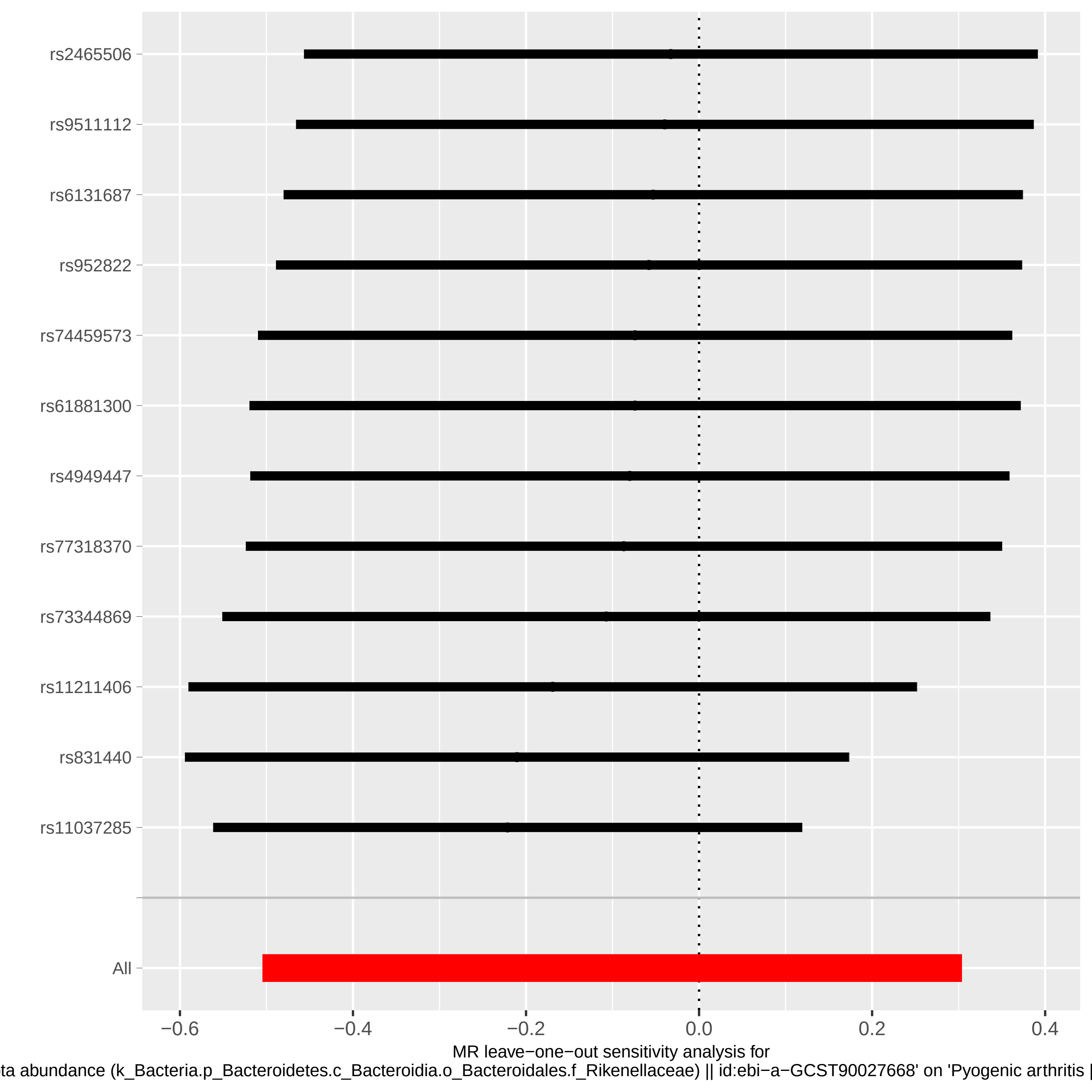

rs2465506
rs9511112
rs6131687
rs952822
rs74459573
rs61881300
rs4949447
rs77318370
rs73344869
rs11211406
rs831440
rs11037285
All
−0.6
−0.4
−0.2
0.0
0.2
0.4
MR leave−one−out sensitivity analysis for
ta abundance (k_Bacteria.p_Bacteroidetes.c_Bacteroidia.o_Bacteroidales.f_Rikenellaceae) || id:ebi−a−GCST90027668' on 'Pyogenic arthritis |
